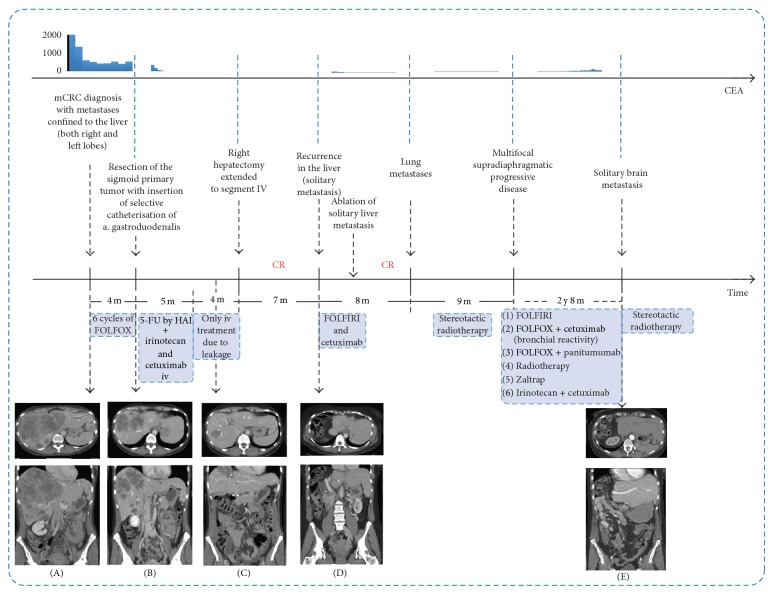# A Case Report of Long-Term Survival following Hepatic Arterial Infusion of *L*-Folinic Acid Modulated 5-Fluorouracil Combined with Intravenous Irinotecan and Cetuximab Followed by Hepatectomy in a Patient with Initially Unresectable Colorectal Liver Metastases

**DOI:** 10.1155/2015/472037

**Published:** 2015-05-06

**Authors:** Kobe Van Bael, Yanina Jansen, Teofila Seremet, Benedikt Engels, Georges Delvaux, Bart Neyns

**Affiliations:** ^1^Department of Surgery, Laarbeeklaan 101, 1090 Brussels, Belgium; ^2^Department of Medical Oncology, Laarbeeklaan 101, 1090 Brussels, Belgium; ^3^Department of Radiotherapy, Laarbeeklaan 101, 1090 Brussels, Belgium

## Abstract

A 43-year-old women admitted to our hospital for weight loss, anorexia, and abdominal pain was diagnosed with sigmoid neoplasm and multiple bilobar liver metastases. This patient received six cycles of systemic FOLFOX prior to a laparoscopically assisted anterior resection of the rectosigmoid for a poorly differentiated invasive adenocarcinoma T2N2M1, K-RAS negative (wild type). Hepatic arterial infusion (HAI) of *L*-folinic acid modulated 5-fluorouracil (LV/5-FU) with intravenous (iv) irinotecan (FOLFIRI) and cetuximab as adjuvant therapy resulted in a complete metabolic response (CR) with CEA normalization. A right hepatectomy extended to segment IV was performed resulting in (FDG-)PET negative remission for 7 months. Solitary intrahepatic recurrence was effectively managed by local radiofrequent ablation following 6c FOLFIRI plus cetuximab iv. Multiple lung lesions and recurrence of pulmonary and local lymph node metastases were successfully treated with fractionated stereotactic radiotherapy (50 Gy) and iv LV/5-FU/oxaliplatin (FOLFOX) plus cetuximab finally switched to panitumumab with CR as a result. At present the patient is in persistent complete remission of her stage IV colorectal cancer, more than 5 years after initial diagnosis of the advanced disease. Multidisciplinary treatment with HAI of chemotherapy (LV/5-FU + CPT-11) plus EGFR-inhibitor can achieve CR of complex unresectable LM and can even result in hepatectomy with possible long-term survival.

## 1. Introduction

The 5-year survival rate of patients with advanced colorectal cancer (CRC) that has metastasized to the liver is determined by the possibility of resection of the primary tumor and liver metastases (LM). Up to 50% of patients with CRC develop LM synchronously or metachronously during the course of their disease. In a majority of stage IV CRC patients, the LM determines the life expectancy [1].

Complete resection of colorectal hepatic metastases, when possible (10–20% of patients presenting with LM), is a standard of care as it can offer the only potential for cure and improve the long-term survival. Reported 5-year survival rates after hepatectomy for CRC-LM are in the order of 27 to 40% [2]. Protoadjuvant treatment with the FOLFOX4 chemotherapy regimen improves the disease-free survival (PFS) rate in patients who are eligible for upfront hepatectomy. There was no difference in the overall survival (OS) in the patients with perioperative chemotherapy compared with the surgery-only group with resectable CRC-LM [3]. A major challenge represents the group of patients with liver-confined metastases that are unresectable at first diagnosis. Despite the improvement in systemic treatment for metastasized CRC, the 5-year survival rates for these patients are below 10% [4, 5]. Patients who cannot undergo upfront resection of their LM may become eligible for resection following systemic treatment with cytotoxic chemotherapy and biological agents targeting the epidermal growth factor receptor (EGFR; cetuximab or panitumumab) or vascular growth factor (VEGF; bevacizumab or aflibercept) (bio)chemotherapy. Over the past few years the so-called downstaging of CRC-LM has been an intense focus of clinical research. Series reported in the literature have suggested that the outcome of patients who become amenable for hepatectomy following response to systemic (bio)chemotherapy is at the level of patients who can undergo upfront hepatectomy resulting in sparking enthusiasm for this sequential therapy approach. However, controversy exists regarding the optimal (bio)chemotherapy regimen to be used and the duration of treatment. In particular the concern for chemotherapy mediated toxicity to the liver compromising the residual functional liver capacity following hepatectomy has been raised [6].

Contemporary neoadjuvant systemic (bio)chemotherapy allows for resection of initially unresectable CRC-LM in about 5–22% of patients. Despite a high rate of recurrence, 5- and 10-year overall survival are, respectively, 33% and 23% with a wide use of repeat hepatectomies and extrahepatic resections (median survival of 39 months) [7]. This strategy has become incorporated in the treatment of CRC with LM, although randomized studies that demonstrate the superiority of this approach are lacking.

With the increasing number of patients with LM receiving surgery, another question concerning the discrepancy between a complete radiologic response and a complete pathologic clearance of cancer comes up. In up to 83% of cases, patients with a complete radiologic response are not found to be in a complete pathologic response following chemotherapy for CRC-LM [8]. This makes the timing for hepatic resection a controversial issue with a high risk of missing “dormant” LM that will progress after the discontinuing the chemotherapy. An Expert Consensus Statement recommends that hepatic metastases should be resected directly when they become resectable [9].

Hepatic arterial chemotherapy infusion (HAI) using fluoropyrimidines (5-fluorouracil or FUDR) has been widely explored for the treatment of CRC-LM. HAI offers the advantage of achieving a high concentration of cytotoxic agents within the CRC-LM, thus providing higher intraliver response rates of up to 83% [10]. A 2-year survival benefit in patients with unresectable metastasis of 48% versus 37% for HAI over systemic chemotherapy was reported [11]. Because of the added complexity and lack of survival advantage in other randomized trials HAI was not adapted as a standard of care. More recently phase I/II studies have demonstrated the feasibility of combining HAI of 5-FU with systemic administration of irinotecan or oxaliplatin and the EGFR targeted monoclonal antibodies cetuximab or panitumumab in patients with RASwt CRC [12, 13]. Our group reported a promising activity with such an approach. A partial response was documented in 62% with median time to progression of 8.7 months. We though also need to note the association of specific toxicity [14].

We here report on a case where HAI of 5-FU-based chemotherapy could downstage the initially unresectable CRC-LM and rendered the patient amenable for hepatectomy achieving an overall survival of more than 5 years.

## 2. Case Presentation

A 43-year-old woman without any prior disease history was admitted to our institution in March 2008 because of a short-lived history of weight loss, anorexia, nausea, and epigastric pain for only a few weeks. Physical examination revealed a hepatomegaly measuring 7 cm under the right costal border. The patient has no familial history of cancer or polyps. Laboratory data showed a strongly elevated carcinoembryonic antigen (CEA) of 1987 *μ*g/L (normal range 0–3.0 *μ*g/L) and disturbed liver tests (LDH 6017 U/L; AST 245 U/L; ALT 135 U/L; ALP 719 U/L; GGT 712 U/L; bilirubin 1.43). Further investigation by Computed Tomography (CT) presumed a tumor of the sigmoid which was confirmed at colonoscopy (at 15 cm of the anal marge, biopsy indicating a poorly differentiated invasive adenocarcinoma). CT examination also visualized voluminous diffuse liver metastases in both left and right lobes with almost complete involvement of segments 4a, 8, and 7 and smaller lesions in segment 2 with a big spur of the left lobe up to the spleen. No extrahepatic metastases were diagnosed (Figure 1).

Because of the involvement of both liver lobes, the patient was ineligible for hepatectomy and six cycles of systemic chemotherapy according to the FOLFOX regimen (LV modulated 5-fluorouracil + oxaliplatin) were administered.

Following an initially rapid decline of the elevated CEA and LDH during FOLFOX therapy, values stabilized. A partial tumor response was obtained but FDG-PET indicated a persistent metabolic activity in the liver. Furthermore, a grade 3 sensorial polyneuropathy became treatment limiting with respect to the oxaliplatin containing chemotherapy regimen. In June 2008, following multidisciplinary consultation, resecting the primary tumor (laparoscopy assisted anterior resection of the rectosigmoid) and inserting an arterial catheter in the gastroduodenal artery (including cholecystectomy) were decided [15]. Pathological staging indicated a poorly differentiated invasive adenocarcinoma stage pT2N2M1, K-RAS wild type.

Intra-arterial treatment was initiated in July 2008 based on the FOLFIRI scheme (LV modulated 5-FU (2400 mg/m^2^ over 48 hours) by hepatic arterial infusion (HAI) + irinotecan (180 mg/m^2^~300 mg over 90 minutes) by intravenous (iv) infusion biweekly). Cetuximab (400 mg/m^2^ over 2 hours once weekly, iv) was added to the treatment regimen.

After three cycles of therapy the patient experienced multiple episodes of palpitations complicated by a few short-lived syncopes. A 48-hour in-hospital Holter-ECG monitoring during treatment of 5-FU by HAI revealed repetitive episodes of sustained and nonsustained monomorphic ventricular tachycardia (frequency of 220–230 beats per minute) at the fourth cycle. An urgent electrophysiologic exploration was done and the focus of the idiopathic right ventricular outflow tract tachycardia was successfully ablated. After five cycles of 5-FU by HAI, treatment administration had to be switched back to iv-administration because of a leak at the catheter tip (diagnosed after the occurrence of abdominal pain associated with administration of chemotherapy by HAI). At this moment, 4 months after surgery and start of HAI, a partial response according to RECIST was obtained with further regression of remnant LM that were no longer characterized by increased fluoro-18-deoxyglucose (FDG) uptake on PET image (providing evidence of a complete metabolic response). In addition, sustained CEA normalization was documented on repeated measurements. Systemic chemotherapy was continued for another 6 months. In April 2009 after a persistent favorable clinical, biological, and radiological evolution, a right hepatectomy extended to segment IV was performed. The postoperative period was complicated with a small bile leak that was effectively managed by ERCP with mini sphincterotomy and intrahepatic placement of a short prosthesis. Otherwise, postoperative recovery was uneventful without hepatic failure. Anatomopathological examination of the liver showed mainly scar tissue after chemotherapy with rare nests of tumoral cells. A complete metabolic response and CEA remission were maintained in the absence of further therapy until November 2009. Recurrence of a solitary liver metastasis was suggested by an elevation in the CEA and confirmed by FDG-PET. The combination of FOLFIRI plus cetuximab was restarted for six cycles after which a stable disease was documented and a successful radiofrequent ablation was performed in February 2010, obtaining a complete response according to RECIST until June 2010 when FDG-PET revealed extrahepatic progression in the lungs (four lung lesions).

In the following years the patient developed additional lung metastasis treated with the combination of systemic chemotherapy and fractionated radiotherapy. A PET-CT in December 2013 showed solitary brain metastases, confirmed on MRI. We treated this patient with stereotactic radiotherapy. The patient was alive and received further systemic therapy at the latest follow-up in November 2014. No recurrence in the liver was documented.

## 3. Discussion

The therapeutic objectives in patients with CRC and upfront unresectable liver-confined metastases have shifted from palliation to maximization of the chance of resection (including also local ablative therapies such as RFA or cryoablation) by applying preoperative systemic (bio)chemotherapy to downsize the LM. In the case described here with initially unresectable voluminous LM, sequential, multimodal treatment including HAI of chemotherapy achieved over-6-year survival. Remarkably, after more than 5 years, the liver remains disease-free. The role of HAI in the treatment of metastatic CRC remains controversial [16]. A recent phase I study of Kemeny et al. demonstrated the conversion to resectability in 47% (even 57% in chemotherapy-naïve patients) with HAI of FUDR in combination with systemic treatment of oxaliplatin and irinotecan [17, 18]. In a previous manuscript, we also described a patient treated in a phase 1 study who experienced an unexpected durable remission after combined-modality treatment involving HAI for synchronous inoperable LM and a solitary lung metastasis of a rectal adenocarcinoma [19].

The reason for the switch from cetuximab to panitumumab was dual. The patient developed a progressive bronchial hyperreactivity. Incidence of hypersensitivity in panitumumab is lower because of its fully humanized nature of the IgG backbone [20]. Whether or not this adjustment results in higher efficacy cannot be confirmed. But it is now safe to say that panitumumab and cetuximab are equally effective and interchangeable in clinical practice, either as monotherapy or in combination with cytotoxic chemotherapy. A switch in idiotype can potentially generate a renewed activity from an anti-EGFR mAb, but this is not supported by scientific evidence.

Today, costs in public health care are of great concern. General cost of overall treatment is beyond the scope of this case report but the increment in survival with good quality of life obviously justifies the financial burden. As a matter of fact, this treatment allowed the patient to remain economically active.

With this technique and contemporarily available efficient biochemotherapy in combination with a dedicated oncosurgical approach, more patients are likely to be able to benefit from extended survival that did not seem achievable at initial diagnosis. A multidisciplinary approach is necessary to obtain such results.

## Figures and Tables

**Figure 1:**